# 3D Printing and Bioprinting in MEMS Technology

**DOI:** 10.3390/mi8070229

**Published:** 2017-07-21

**Authors:** Chee Kai Chua, Wai Yee Yeong, Jia An

**Affiliations:** Singapore Centre for 3D Printing, School of Mechanical and Aerospace Engineering, Nanyang Technological University, 50 Nanyang Avenue, Singapore 639798, Singapore, WYYeong@ntu.edu.sg (W.Y.Y.); AnJia@ntu.edu.sg (J.A.)

**Keywords:** MEMS, 3D printing, bioprinting, additive manufacturing, rapid prototyping

## Abstract

3D printing and bioprinting have advanced significantly in printing resolution in recent years, which presents a great potential for fabricating small and complex features suitable for microelectromechanical systems (MEMS) with new functionalities. This special issue aims to give a glimpse into the future of this research field.

3D printing, also known as rapid prototyping or additive manufacturing, is a family of layer-by-layer fabrication processes [1]. Some well-established processes for printing polymers include stereolithography [2], polyjet [3], fused deposition modeling [4], selective laser sintering [5], and binder jetting [6]. Other processes typical for powder-based ceramic printing or metal printing include selective laser melting and electron beam melting [7,8,9]. However, since metal printing is relatively new, their process mechanisms are still under investigation [10]. Recent new processes joining the 3D printing family include bioprinting [11,12] and 4D printing [13,14], which expanded the applications of 3D printing towards printing of living materials and smart materials.

All 3D printing technologies share a common process chain, which starts with an advanced custom model in computer, such as NURBS-based volumetric model in the case of biomedical objects [15], followed by converting the model to an interface recognized by 3D printers. A commonly used interface is the stereolithography (STL) model, though there are many other interfaces available [16]. Generally, the STL model is checked for errant faces before being sliced into many layers and sent to the 3D printer for fabrication. Depending on the material required in the final application, 3D printed parts can be used directly or indirectly [4,17]. Indirect use means that the part is used as an intermediate mold for the final part. Both direct and indirect approaches could be relevant in making new MEMS devices, subject to the feature size. 

Among all 3D printing processes, inkjet printing and direct laser writing present the highest resolution achievable, with the smallest feature size ranging from a few hundred micrometers down to a few hundred nanometers [18]. This provides a resolution basis for direct or indirect printing of MEMS devices. Regarding materials, direct laser writing has a similar range compared to inkjet printing [19], but generally inkjet printing is able to print multiple materials in a single layer [20]. Therefore, for MEMS components with ultrafine features, direct laser writing is better, while inkjet printing could be used to print assemblies of multi-material components on various surfaces.

In this short but timely special issue, two reviews and two research articles are included. Lau and Shrestha reviewed current developments in inkjet printing of MEMS devices and they suggested that inkjet printing is well suited for printing two-dimensional or surface MEMS devices from a small unit to an array over a large area [21]. Mao et al. reviewed the principles and the recent advances of high-resolution 3D printing techniques and provided a complete landscape of these exciting developments [22]. Liu et al. presented a study on the effect of 3D nanofiber bundles on the morphology and contraction of cardiac cells [23]. Finally, Tran et al. reported an inkjet-printed alternating current (AC) electrokinetic device with the smallest feature size of 60 μm [24].
